# Mechanisms of spinal cord stimulation for the treatment of pain: Still in the dark after 50 years

**DOI:** 10.1002/ejp.1336

**Published:** 2018-12-03

**Authors:** Melanie P. Jensen, Robert M. Brownstone

**Affiliations:** ^1^ Sobell Department of Neuromuscular Diseases, Institute of Neurology University College London London UK

## Abstract

**Background and Objective:**

Despite the value of spinal cord stimulation (SCS) in treating some patients with focal neuropathic pain, technological advances in stimulator design and treatment protocols have not correlated with significant improvements in clinical outcomes. This may be because incomplete understanding of the mechanisms underlying SCS precludes improvement in clinical efficacy. In this brief review, we (a) review phenomenological effects of SCS, (b) review the literature on proposed spinal sites of action of SCS and (c) propose a novel hypothesis of mechanism of action.

**Results:**

Dorsal columns, dorsal roots and dorsal horns have each been proposed as spinal sites of action of SCS. We suggest that evidence in favour of the dorsal columns or dorsal roots as the primary mediators of SCS is weak and propose that the dorsal horn is the crucial site of action. Furthermore, we hypothesize that, based on their location, and neurochemical and morphological properties, dorsal horn islet cells may mediate the effects of SCS.

**Conclusions:**

The precise spinal mechanisms of action of SCS are still unknown. Dorsal horn islet cells have properties that position them to play a key role in analgesic effects of electrical stimulation. Understanding the mechanisms responsible for positive SCS effects are needed for successful translation into clinical dividends.

**Significance:**

We review possible spinal mechanisms of action of spinal cord stimulation for neuropathic pain, proposing that direct modulation of dorsal horn neurons is crucial. We suggest that mechanistic insights are needed for translation into more favourable clinical outcomes.

## 1 INTRODUCTION

Spinal cord stimulation (SCS) was first reported as a treatment for pain a half‐century ago (Shealy, Taslitz et al., 1967). Since then, this use of electrical stimulation via leads placed in the spinal dorsal epidural space has become a valuable therapeutic tool for treating neuropathic pain. The field of neuromodulation for chronic pain is rapidly expanding: in recent years, over 25,000 neurostimulators have been implanted annually in the United States alone (Prager, 2010). While the economics points towards cost‐effectiveness of SCS (Kumar and Rizvi, 2013), the price of SCS devices is increasing. Furthermore, concomitant technological “advances”, including complex stimulator designs and treatment protocols, have not correlated with improvements in patient outcomes (Zhang et al., 2014). This stalling of clinical efficacy perhaps indicates that we have reached an absolute asymptote in the capacity of SCS therapy to improve quality of life. Here, however, we suggest that it is our incomplete understanding of the mechanisms of SCS that has prevented further advancement.

If, as in drug discovery, high quality mechanistic insights lead to improved therapies (Howick et al., 2010), it would be useful to understand the mechanisms of action of SCS in modulating neuropathic pain. Here, we focus on potential spinal sites of action—that is, what is happening at the site of therapy delivery—recognizing that supraspinal mechanisms also contribute to pain reduction (Bantli et al., 1975; Linderoth and Foreman, 1999). Furthermore, we will focus on conventional stimulation therapy, given the half century of experience with this treatment modality compared to the limited data on new SCS algorithms, such as high frequency and burst stimulation (Linderoth and Foreman, 2017). We first outline some physiological effects of SCS, then present evidence against previously hypothesized sites of action: dorsal columns and dorsal roots. We then propose the dorsal horn as the likely key site of action, and finally hypothesize that SCS stimulates dorsal horn islet cells to reduce neuropathic pain.

### 1.1 Neurophysiological and neurochemical phenomenology of SCS

It has been proposed that the therapeutic benefit of SCS results, in part, from changes in cortical activity: after all, pain is experienced by the brain. The supraspinal effects of SCS have been explored using imaging techniques. fMRI studies have shown that SCS leads to increases in activation of primary and secondary sensorimotor and posterior insular cortices (Stancak et al., 2008), and changes in “functional connectivity” between sensory and limbic areas (Deogaonkar et al., 2016). ^15^H_2_O PET studies have shown an increase in blood flow to the thalamus, bilateral parietal association areas, anterior cingulate cortex, and prefrontal areas with SCS (Kishima et al., 2010). These results have led to the suggestion that the cortical effects of SCS may down‐regulate the negative affective components of pain and modulate pain thresholds (Stancak et al., 2008; Kishima et al., 2010; Bentley et al., 2016). However, methodological variability, clinical heterogeneity across cohorts, and the diversity of cortical changes in response to SCS limits the robustness of conclusions; a recent systematic review highlighted the paucity of conclusive mechanistic insights relating to supraspinal effects (Bentley et al., 2016). Critically, it is unclear whether the effects seen in the cortex reflect a top‐down, cortical‐spinal process to mediate analgesia, or rather responses to a therapeutic effect initiated at the spinal level (a bottom‐up, spinal‐cortical mechanism), or a combination of the two.

In the spinal cord, electrophysiological studies have demonstrated that that there is an attenuation of nociceptive reflexes with SCS (Garcia‐Larrea et al., 1999; de Andrade et al., 2010), as well as some changes to sympathetic reflexes, a reduction in H‐reflex, and a reduction in a component of the somatosensory evoked potentials (de Andrade et al., 2010; Wolter et al., 2013). Interestingly, analgesia induced by SCS correlates with nociceptive reflex attenuation, interpreted as SCS causing broad inhibition of sensory afferent inputs (de Andrade et al., 2010), but any mechanistic relationship between reflex reduction and pain relief is not clear.

It has also been shown that SCS leads to neurochemical changes. These insights have largely come from animal models, where antagonists to GABA, serotonin, noradrenaline and dopamine each reduced some effects of SCS (Barchini et al., 2012). For example, the suppression of tactile allodynia by SCS was found to be associated with GABA release in the dorsal horn (Stiller et al., 1996). A recent study showed that SCS led to reduced dorsal horn expression of an NMDA receptor subtype typically associated with peripheral sensitisation (Sun et al., 2017). In the same study, CB1 receptors were found to be responsible for the reversal of hyperalgesia seen with SCS (Sun et al., 2017). But again, any mechanistic relationship between SCS and these neurochemical changes is unknown.

Thus, it is clear that SCS can lead to pain reduction, ultimately seen as changes in brain activity, and that SCS leads to neurochemical and physiological changes in the spinal cord. But the key mechanistic question remains: How does electrical stimulation of the spinal cord lead to analgesia?

### 1.2 Dorsal column stimulation?

Spinal cord stimulation was developed as a direct spin off of the gate control theory of pain (Melzack and Wall, 1965). Melzack and Wall postulated that afferent pain signals, transmitted to the spinal cord via small fibres, are opposed by simultaneous activation of cutaneous touch signals, transmitted via larger myelinated fibres. This low threshold cutaneous activity would result in the “gating” of dorsal horn output, thus reducing central pain perception. SCS stemmed from this theory: it was postulated that continuous stimulation of the axonal branches of Aβ fibres in the dorsal columns would lead to transmitter release via their spinal collaterals, which would then lead to inhibition of C fibre responses in dorsal horn neurons. This would close the gate and reduce central transmission of pain signals (Shealy, Taslitz et al., 1967; Wall and Sweet, 1967). This concept led to the initial term “dorsal column stimulation” (Shealy, Mortimer et al., 1967).

Clinical data lent indirect support to this theory: SCS induces regional paraesthesias, thought to be via stimulation of large cutaneous afferents, and coverage of the painful area by paraesthesias is thought to be necessary for therapeutic effect (Barolat et al., 1993; North and Roark, 1995; Holsheimer, 1998). This is consistent with the notion that SCS stimulates Aβ afferent fibres in the dorsal columns.

However, evidence suggests that the mechanisms of SCS are more nuanced. In particular, SCS primarily modulates chronic neuropathic pain, doing little to alleviate acute nociceptive pain as would be predicted from the gate control theory (Shealy, Mortimer et al., 1967; Shealy, Taslitz et al., 1967). That is, this evidence conflicts with the concept that SCS acts simply through stimulation of large afferent fibres that would result in gating transmission from nociceptive afferents.

But the concept that direct stimulation of the dorsal columns leads to analgesia is topographically appealing: dorsal columns are the intraspinal structures nearest to the electrodes, so it is logical that they be stimulated by a midline epidural electrode (Holsheimer et al., 1995; Holsheimer, 1998). Conceptually, this made sense: the extent to which a neuron or axon is excited by stimulation‐induced electric fields depends not only on proximity to the stimulating electrode, but also on the structure’s electrical properties. Dorsal columns are longitudinal white matter tracts that should be activated by a longitudinally positioned cathode/anode combination (Oakley and Prager, 1976; Struijk et al., 1992; Brocker and Grill, 2013). And in animal models, sectioning of the dorsal column rostral to the site of stimulation leads to a reduction (although not elimination) in SCS effectiveness, suggesting that longitudinal fibres (of primary afferents or postsynaptic dorsal column pathways) play a role, along with modulation of the spinal cord itself which produces dorsal column‐independent analgesia (Barchini et al., 2012). But given the presence of postsynaptic dorsal column pathways (Rustioni, 1973), the analgesic effects mediated by the dorsal columns may not be mediated by their direct stimulation.

Furthermore, the dorsal column stimulation hypothesis falls short with respect to two neuroanatomical properties. Firstly, dorsal column fibres are arranged such that the fasciculus gracilis (which transmits afferent signals from the lower limbs) lies medial to the fasciculus cuneatus (which transmits afferent signals from the upper limbs) (Smith and Deacon, 1984). If SCS functions via stimulation of the dorsal columns, cervical SCS via a midline stimulating electrode should elicit paraesthesias in the lower limbs at lower thresholds than those needed to elicit paraesthesias in the upper limbs. Instead, as stimulation amplitude is increased, paraesthesias are produced first bilaterally in the arms and then spread caudally (Meyerson, 1975). This indicates that if paraesthesias are produced by stimulation of the dorsal columns, then the lateral dorsal columns are stimulated at lower current intensities than the medially placed, nearer‐to‐lead fasciculus gracilis, an unlikely geometric condition. Secondly, given that dorsal column fibres run longitudinally, small adjustments in rostro‐caudal position of stimulation would result in a relatively small change in the number of fibres being stimulated. Yet, the most favourable response is achieved by a relatively localized array of stimulating electrodes, with small rostral or caudal adjustments altering paraesthesias and clinical effects (Holsheimer, 1998). Thus, the anatomical geometry indicates that it is unlikely that the effects of SCS arise primarily from dorsal column stimulation.

### 1.3 Dorsal root stimulation?

Given the positional sensitivity of the effects of SCS, it is logical to ask whether dorsal roots could be the primary site of action of SCS (Struijk et al., 1993). Modelling studies predict that the ventral curvature of incoming dorsal root afferents should decrease their stimulation thresholds (Coburn and Sin, 1985; Struijk et al., 1993). Large myelinated dorsal root fibres have low stimulation thresholds and may be recruited prior to the dorsal columns by stimulation‐induced electrical fields (Struijk et al., 1993). While logical, several pieces of evidence suggest that dorsal root stimulation is unlikely to be clinically prominent in achieving therapeutic effects.

Firstly, when ramping up the amplitude of SCS, there is a large range in paraesthesia‐producing amplitudes prior to reaching the threshold for producing motor effects. But group Ia muscle spindle afferent fibres, which enter the spinal cord in a medial position, close to the position of SCS leads, have lower (or possibly similar in humans (Macefield et al., 1989)) thresholds compared to the largest, lowest threshold Aβ cutaneous fibres. If the effects of SCS are mediated by stimulation of dorsal root afferents as they enter the cord and curve ventrally, then at low thresholds group Ia afferents should be activated leading to muscle contractions via monosynaptic reflex pathways. In addition, one would expect Ia afferent stimulation to produce proprioceptive errors. Yet, SCS typically elicits paraesthesias over a large range of stimulus amplitudes that are lower than those required to produce motor responses mediated by monosynaptic reflex pathways, and proprioceptive errors do not appear to be an effect of SCS (Rijken et al., 2013), making it unlikely that SCS functions by stimulating dorsal root afferents.

Secondly, focal SCS leads to paraesthesias in more than one dermatome—for example, a whole leg, indicating that it is not only local afferent fibres that are stimulated. While deviation of electrodes laterally can be used to directly stimulate individual dorsal roots, this produces dermatomal paraesthesias, which may result in focal analgesia. In addition, the paraesthesias produced by direct dorsal root stimulation occur at stimulation amplitudes much lower than those required to produce paraesthesias by midline stimulation (Oakley and Prager, 1976; Barolat et al., 1993; He et al., 1994). Together, these differences indicate that the mechanism(s) underlying the usual midline or paramedian SCS therapy is unlikely to be mediated by dorsal root stimulation alone.

This does raise the question, though, of whether presynaptic inhibition (PSI) of afferent fibres may play a role in the analgesic effects of SCS, as has been proposed (Shimoji et al., 1982). PSI is depressed in models of neuropathic pain (Guo and Hu, 2014), so it is logical to ask whether SCS acts to “restore” PSI and thus reduce nociceptive input to the nervous system. But given that classical PSI requires stimulation of afferents, either in the dorsal columns or dorsal roots, it seems, based on the above, to be an unlikely primary mechanism of action. In addition, PSI occurs in direct response to stimulation and with a very short (<1 s) time course (Eccles et al., 1963), whereas the analgesic effects of SCS build up over minutes and continue following cessation of SCS (Oakley and Prager, 1976). While it is therefore unlikely that SCS acts via stimulating afferents to simply “restore” PSI–mediated gating of nociceptive afferent transmission, it is possible that the stimulation produces plastic changes (see Rudomin, 2009) in presynaptic inhibitory neurons that alter pain transmission (Wall and Sweet, 1967).

### 1.4 Dorsal horn stimulation?

The dorsal horn is a critical site of initial pain processing and much focus has been on its role in neuropathic pain (Costigan et al., 2009). Neuronal circuits in the dorsal horn act as processing hubs, analysing incoming sensory information for appropriate onward transmission to projecting neurons. It has been suggested that since midline epidural electrodes ensure the stimulation‐generated electrical fields would be greater in the superficial dorsal horn than in the adjacent dorsal root, this is the preferred target for SCS (Oakley and Prager, 1976). It is thus logical to ask whether the dorsal horn could be the primary site of action of SCS.

In rodent models of neuropathic pain displaying allodynia after sciatic nerve ligation, neurons of the deep dorsal horn (in particular wide dynamic range neurons and nociceptive‐specific neurons) exhibit hyperexcitability (Laird and Bennett, 1993). SCS depresses hyperexcitability of wide dynamic range neurons of the dorsal horn, dampening both increased frequency of spontaneous discharge to cutaneous stimuli and after‐discharge (Yakhnitsa et al., 1999). This effect is long‐lasting (over 10 min) and often peaks after the cessation of SCS, correlating with the clinical observation that relief afforded by SCS can outlast the period of stimulation (Wall and Sweet, 1967). It is not clear whether the inhibitory action of SCS on hyper‐excitable wide dynamic range neurons is mediated via reducing their excitatory input, increasing their inhibitory input, modulating the electrophysiological properties of these neurons, or a combination of these effects.

Animal models of neuropathic pain provide evidence that dysfunction of superficial dorsal horn inhibitory interneurons, with resultant decreases in inhibition, contribute to neuropathic pain, including allodynia and hyperalgesia (for review, see Todd, 2010). Such a reduction in inhibition could lead to the hyperexcitability seen in wide dynamic range neurons. In rodent models of neuropathic pain, there are changes in local circuit function in the superficial dorsal horn as shown, for example, by a reduction in afferent‐evoked inhibitory postsynaptic currents in lamina II interneurons (Moore et al., 2002). Whether this circuit dysfunction results from a loss of inhibitory interneurons, reduced tonic excitation of inhibitory neurons (Balasubramanyan et al., 2006), depletion of GABA, or abnormal postsynaptic responses to GABA is not clear (Ibuki et al., 1997; Moore et al., 2002). In favour of the latter, the potassium chloride cotransporter KCC2, a regulator of intracellular chloride levels, is down‐regulated in rodent models of neuropathic pain such that GABA induces depolarizing rather than hyperpolarising responses, leading to neuronal excitation (Coull et al., 2003). Whichever the process(es), the specific identity of neurons involved is not known, although evidence points towards superficial dorsal horn inhibitory interneurons. Given that epidural stimulation (at 1–2 Hz) has been shown to activate inhibitory interneurons of lamina I–III—albeit with latencies consistent with trans‐synaptic (i.e. indirect) activation (Dubuisson, 1989)—it seems logical to pursue the question of whether stimulation (at therapeutic frequencies) of inhibitory neurons in this region is the main mechanism underlying the therapeutic benefit of SCS.

**Figure 1:**
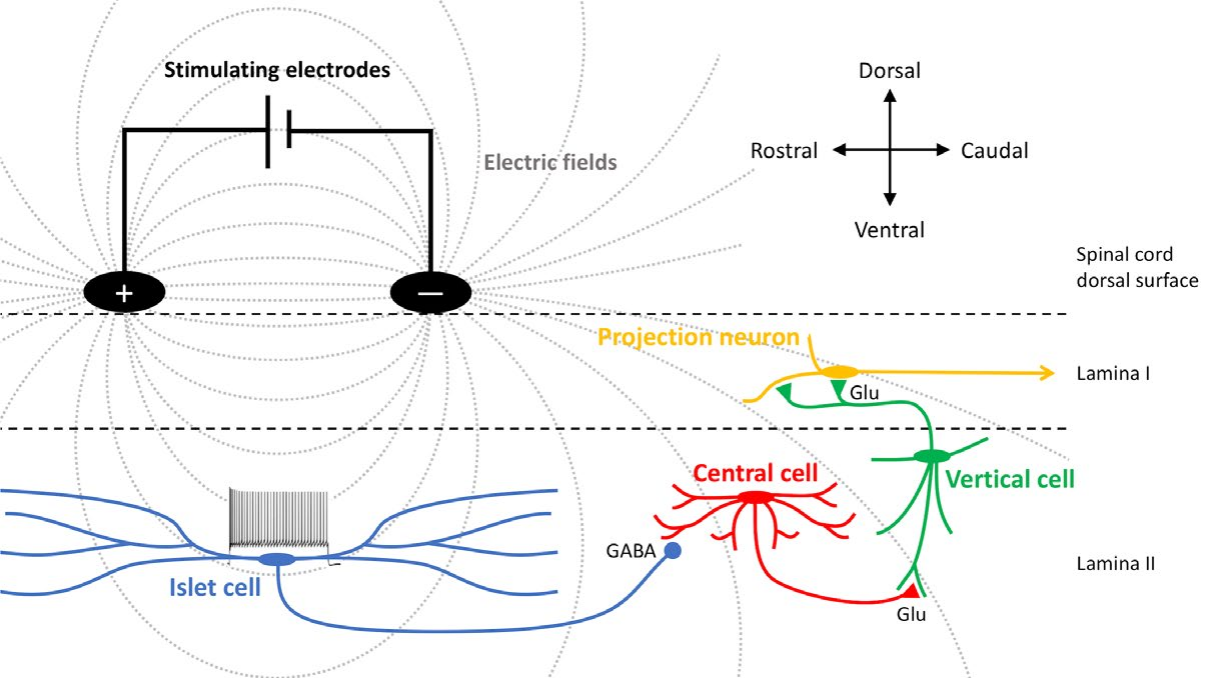
Schematic illustrating proposed mechanism of action of spinal cord stimulation for pain. Surface stimulation (black electrodes) produces electric fields (grey lines) that span dorsal horn islet cells (blue) leading to activation of their dendrites, depolarization, and thus trains of action potentials. Islet cells in turn would inhibit transmission between excitatory interneurons (shown as central cells, red and vertical cells, green), which would result in reduced activity of projection neurons (shown as lamina I projection neurons, yellow). (cf. Lu and Perl, 2005; Todd, 2017).

One population of neurons in the superficial dorsal horn deserves particular attention: islet cells. Islet cells in the superficial dorsal horn have long dendritic trees (>400 μm) oriented in the rostro‐caudal direction with minimal mediolateral or dorsoventral projections (Todd and Lewis, 1986; Lu and Perl, 2003; Yasaka et al., 2007). As islet cells are GABAergic (Todd, 2010), it is plausible that their dysfunction could lead to the reduction in inhibition seen in neuropathic pain models. Furthermore, islet cells are tonically firing neurons, owing to their relatively depolarized resting membrane potential (Lu and Perl, 2005). This is consistent with a postulated circuit in which islet cells tonically inhibit central cells in lamina II that form excitatory synapses with vertical cells, which in turn innervate projecting neurons of lamina I that mediate pain signals (Lu and Perl, 2003; 2005) (Figure 1). In neuropathic pain, there is a reduction in excitatory input to islet cells (Balasubramanyan et al., 2006), which could result in reduced islet cell‐mediated transmission, and consequently lead to disinhibition of excitatory interneurons responsible for transmitting nociceptive signals (Todd, 2010).

Having said that, it is not clear what the target neurons of islet cells are, nor whether they are responsible for pre‐ or postsynaptic inhibition of their targets. It is known that some parvalbumin‐expressing neurons in the dorsal horn form axo‐axonic synapses (mediating presynaptic inhibition) with fibres from low threshold mechanoreceptors, and some islet cells do express parvalbumin (Hughes et al., 2012). And there is also evidence that islet cell axons form axo‐dendritic synapses (mediating postsynaptic inhibition) with neurons of the superficial dorsal horn (Gobel et al., 1980). Knowledge of the synapses formed by islet cell axons is necessary for understanding the role(s) of these neurons in neuropathic pain.

## 2 Hypothesis: SCS activates islet cell dendrites

In considering possible mechanisms of SCS, it is useful to examine how electrical fields produced by stimulation might affect neurons with large, polarized dendritic trees. These effects can be considered in light of studies using intracellular recordings from motoneurons with polarized dendritic trees in *in vitro* preparations of turtle spinal cords (Hounsgaard and Kiehn, 1993). In those studies, applying electrical fields across the longitudinal axes of dendrites led to depolarization of distal dendrites, generating calcium currents that led to sustained firing activity in the somata (Hounsgaard and Kiehn, 1993). Thus, electrical fields can have profound effects on activity and integration in morphologically complex neurons with active dendrites, and these effects critically depend on the trajectory of dendrites in relation to the direction and geometry of the applied current (Jankowska, 2017).

As noted above, the dendrites of islet cells are oriented in the rostro‐caudal direction such that they would be spanned by SCS electrodes (Figure1). We suggest here that SCS mediates its therapeutic effect by exciting islet cell dendrites, thus increasing the spiking of islet cells. As such, SCS may “compensate” for the reduced excitation of islet cells seen in neuropathic pain models (Balasubramanyan et al., 2006). This concept is consistent with the demonstration that in neuropathic pain models that produce signs of tactile allodynia in rodents, intrathecally administered GABA agonists augment withdrawal thresholds and restore responses to SCS (Cui et al., 1996). Furthermore, in animal models of SCS, stimulation leads to an augmentation of GABA and reduction in glutamate release in the dorsal horn (Cui et al., 1997), and there is a reduction in excitability of wide dynamic range neurons (Guan et al., 2010), putative synaptic targets of islet cells (Todd, 2010). That is, SCS may restore inhibition by enhancing dorsal horn GABAergic systems. We suggest that, given the geometry of stimulation, a prime candidate for these effects would be islet cells.

Interestingly, in patients with SCS, when the pulse width (duration) of stimulation is increased, the area of paraesthesias increases (Holsheimer et al., 2011). This indicates that structures with higher chronaxies – either because of their position in relation to the electrode or their intrinsic properties – are then recruited. It is known that passive dendrites have significantly longer chronaxies than axons (Rattay et al., 2012), raising the possibility that the effects of increasing pulse widths result from increased recruitment of dendrites. In fact, longer pulse widths may preferentially recruit dendrites even in passive neurons (Stern et al., 2015). And if islet cell dendrites have active properties (voltage‐gated channels), it is likely that they would have lower chronaxies than passive dendrites (Stern et al., 2015), so a proportion, for example, would be stimulated with relatively low pulse widths. Thus the clinical effects observed when increasing pulse widths could be explained by increasing activation of islet cell dendrites.

Finally, can this hypothesized mechanism explain the time course of the effects of SCS, which can produce analgesic effects that outlast the duration of stimulation (Oakley and Prager, 1976)? As in other regions of the nervous system, repetitive stimulation of spinal neurons can lead to long‐lasting changes in their activity (Mendell, 1984). In fact, it has been shown that there are plastic changes in the dorsal horn in a number of pain models (Todd, 2015). Can the repetitive stimulation of SCS lead to a “reversal” of these changes? It is conceivable that the hyperexcitability of some dorsal horn excitatory neurons associated with pain can be reduced by SCS‐evoked enhancement of inhibition mediated by islet cells, and that this persistent inhibition leads to plastic changes and a reduction in excitability, thus reducing pain (Hu et al., 2006; Yasaka et al., 2010). These plastic changes would then outlast the electrical stimulation, thus explaining the time course of the clinical effects of SCS.

In summary, by correlating our understanding of the effects of electrical fields on neuronal excitability with dendritic morphology and known or postulated neuropathic pain mechanisms, we hypothesize that SCS works by stimulating dendrites of dorsal horn islet neurons. There are many unanswered questions raised by this hypothesis that can start to be addressed using methodologies in rodents once islet cells can be genetically targeted (like other spinal cord neurons, e.g. Chopek et al., 2018). For example, does SCS lead to stimulation of islet cells? This could be studied by recording genetically identified islet cells and applying external electrical fields. Does islet cell excitation lead to analgesia? This could be studied using targeted stimulation (optogenetic or chemogenetic) of islet cells in animal models of neuropathic pain. Causality may then be inferred if: (a) directly activating islet cells mediates analgesia (demonstrating sufficiency) and (b) selectively inhibiting islet cells abolishes the therapeutic effects of SCS (demonstrating necessity). Having said that, such experiments will fail to capture the uniquely human affective‐motivational component of pain. That is, manifestations of “therapeutic effect” in animal models of neuropathic pain may not translate to humans (Meyerson and Linderoth, 2006). But identifying the spinal site of action of SCS will no doubt clarify how SCS modulates the sensory component of pain. And understanding the mechanisms by which electricity impacts surrounding structures is the next logical step to advancing neuromodulation therapy.

## 3 CONCLUSION

The general phenomenology of neuropathic pain in patients and the therapeutic effects and limits of SCS for the treatment of neuropathic pain have been well described. To advance electrical stimulation therapies further, animal models aimed at studying neuronal circuits underlying neuropathic pain and how these circuits are modulated by SCS will undoubtedly provide invaluable understanding (see Linderoth and Foreman, 2017). Taking knowledge from phenomenological studies in patients to mechanistic studies in animal models would logically lead to the development of mechanism‐based interventions that may lead to clinical gains in treating neuropathic pain in patients: a bedside to bench to bedside approach. That is, by moving from Platonic inductive reasoning of modelling to Aristotelian deductive reasoning of experimentation, we may ultimately understand the mechanisms of this therapy. Sound mechanistic insights have proved pivotal in improving pharmacological treatments (Howick et al., 2010). Applying mechanistic approaches to neurostimulation therapies will lead to real advances in SCS therapies that translate into clinical dividends. We could then emerge from this half‐century‐long impasse.

## CONFLICTS OF INTEREST

None.

## AUTHOR CONTRIBUTIONS

RMB and MPJ worked together to identify studies relevant to the topic and draft the manuscript. RMB designed the study concept, and critically revised the manuscript.
